# Lowering blood pressure is a priority in Brazil and worldwide

**DOI:** 10.1590/1516-3180.2017.1356211117

**Published:** 2017-09-28

**Authors:** Paulo Andrade Lotufo

**Affiliations:** I MD, DrPH. Full Professor, Department of Internal Medicine, Faculdade de Medicina da Universidade de São Paulo (FMUSP), São Paulo (SP), Brazil.

In November 2017, the American Heart Association (AHA) and the American College of Cardiology (ACC) launched new guidelines for detecting, treating and controlling hypertension.^1^ Recommendations from US researchers have been reaching significant broad audiences through several guidelines that have been disseminated worldwide. Starting in the 1970s, the US National Institutes of Health (NIH) issued seven guidelines relating to management of high blood pressure^2^. However, in 2014, the NIH declined to issue new instructions and asked these American professional associations to update the approach to hypertension.

The cornerstone of the new guidelines has come from the results of the Systolic Blood Pressure Intervention Trial (SPRINT). This was a large randomized clinical trial that revealed that the risks of all causes of deaths and of new cases of cardiovascular diseases were lower only at blood pressure levels lower than the previous cutoff of 140 mmHg for systolic blood pressure (SBP) and 90 mm for diastolic blood pressure (DBP).^3^

The data from SPRINT reaffirmed what had been seen in several observational studies that showed that people with prehypertension were at higher risk. The most relevant data had come from the Framingham Heart Study in 2001: the 10-year cumulative incidence of cardiovascular disease among people with prehypertensive blood pressure levels (SBP of 130 to 139 mmHg, DBP of 85 to 89 mmHg, or both) in the age range from 35 to 64 years was 4% (women) and 8% (men); and at ages of 65 years and over, the incidence was 18% (women) and 25% (men).^4^

Briefly, based on risk findings and clinical trial experience, these guidelines recommend that for individuals with average SBP ≥ 130 mmHg or average DBP ≥ 80 mmHg, accurate outpatient clinic and home blood pressure monitoring measurements are needed. Use of drugs after the diagnosis is recommended for everyone with SBP ≥ 140 mmHg or DBP ≥ 90 mmHg. Among people presenting SBP values ranging from 130 to 139 and DBP from 80 to 89, use of medicines is recommended for those with high cardiovascular risk, diabetes, chronic kidney disease or previous cardiovascular diseases.^1^

From our point of view, there are two essential points to be discussed with all physicians and healthcare providers in Brazil.

1. The diagnosis of hypertension does not need to be immediate. A sequence of blood pressure measurements taken at home or in an ambulatory setting should be the gold standard, and not the office blood pressure. At first glance, the impression obtained is that the range of blood pressures that are deemed to be hypertensive has been significantly increased. However, the spread of home and ambulatory blood pressure measurements will reduce the level of values obtained, in comparison with the blood pressure measured in the physician’s office, which was the source of the survey data addressing hypertension.
2. The aim in lowering high blood pressure is to reduce the incidence and recurrence of cardiovascular diseases. Consequently, a general approach to risk factors is fundamental for all patients and mandatory for people who are tagged with the diagnosis of hypertension. Thus, the need to calculate the overall risk will demand information about smoking, diabetes, renal function and previous cardiovascular diseases.

It is always necessary to remember that the most crucial issue regarding lowering of blood pressure to curb the burden of cardiovascular diseases relates to primordial prevention. Thus, there remains the need to reduce the high intake of salt in Brazil,^5^ and the high mortality rate among people with previous cardiovascular conditions that arises from the lack of secondary prevention in this country.^6^

It now becomes necessary for the previous analyses in several Brazilian epidemiological studies to be rerun. These studies include the Brazilian Longitudinal Study of Adult Health (ELSA-Brasil),^7^ Bambuí Cohort Study of Aging, ^8^ Pelotas (Brazil) Birth Cohort Study,^9^ Prospective Urban Rural Epidemiology,^10^ First National Survey of Indigenous People’s Health and Nutrition^11^ and 2013 National Health Survey.^12^ The aim in so doing will be to identify the impact of the 2017 ACC/AHA high blood pressure guidelines, in accordance with the different population subsets evaluated by those studies.
